# The changes of rhizosphere microbial communities in pepper varieties with different capsaicinoids

**DOI:** 10.3389/fmicb.2024.1430682

**Published:** 2024-08-26

**Authors:** Xin Li, Yan Zhang, Chi Zhou, Xuefeng Li, Xuexiao Zou, Lijun Ou, Yu Tao

**Affiliations:** ^1^Institute of Vegetable, Hunan Academy of Agricultural Sciences, Changsha, Hunan, China; ^2^Institute of Vegetables and Flowers, Chinese Academy of Agricultural Sciences, Beijing, China; ^3^Key Laboratory for Vegetable Biology of Hunan Province, College of Horticulture, Hunan Agricultural University, Changsha, Hunan, China

**Keywords:** pepper, varieties, capsaicinoids, microbial community, rhizosphere

## Abstract

Capsaicinoids are produced uniquely in pepper fruits, and its level determines the commercial quality and health-promoting properties of pepper. So, it is particularly important to increase capsaicinoids content in pepper. Rhizosphere microbiota is critical to plant growth and performance, and affected by plant varieties. However, the impact of pepper varieties with different capsaicinoids yields on the rhizosphere microbiota is poorly understood. Using high-throughput sequencing of the 16S rRNA and internal transcribed spacer (ITS) region, we investigated the rhizosphere microbial community among five pepper varieties containing different capsaicinoids. Our results demonstrated that pepper variety significantly influenced the diversity and structure of rhizosphere microbial community. Bacterial diversity in varieties with high capsaicinoids content was significantly higher than in varieties with low capsaicinoids content, while fungal diversity was opposite to bacterial diversity. The correlation analysis revealed that 19 dominant bacterial genera (e.g., *Chujaibacter*, *Rhodanobacter*, and *Gemmatimonas*) were significantly correlated with capsaicinoids content, and nine of them were also significantly associated with soil nutrients, whereas only one fungal genus (*Podospora*) was significantly correlated with capsaicinoids content. Additionally, almost all genera which significantly correlated to capsaicinoids content were biomarkers of the five pepper varieties and the correlation was well corresponding to the capsaicinoids content. Overall, our results confirmed that the variety of pepper significantly affected the rhizosphere microbial community in the fields, and bacteria and fungi responded differently to capsaicinoids, which may affect the biosynthesis of capsaicinoids and contribute to further improvement of capsaicinoids production in pepper fruits.

## Introduction

Pepper (*Capsicum*) is native to the tropical and temperate Americas (Carrizo García et al., 2016). The genus *Capsicum* contains approximately 35 species, of which the major five cultivated species are *Capsicum annuum*, *Capsicum baccatum*, *Capsicum chinense*, *Capsicum frutescens*, and *Capsicum pubescens*. Among them, *Capsicum annuum* is the most widely cultivated species in the world-wide (Pickersgill, 1997; Carrizo García et al., 2016). Pepper displays a special pungency, which is formed by the accumulation of a kind of alkaloids synthesized in the placenta, called capsaicinoids, whose major representatives are capsaicin and dihydrocapsaicin, accounting for almost 90% of all capsaicinoids (Kozukue et al., 2005; Aza-González et al., 2011). Capsaicinoids show the potential applications range from food flavorings to therapeutics. For instance, capsaicinoids have antimicrobial properties and might be useful as biopesticides (Xing et al., 2006). It has been described that capsaicinoids protect *Capsicum chacoense* seeds against *Fusarium*, which is a major cause of predispersal seed mortality (Tewksbury et al., 2008). Additionally, capsaicinoids possess the biological properties of antitumor, antioxidant and anti-obesity, and are used for food, pharmaceutical, medical, cosmetic and dietary (Aza-González et al., 2011; Luo et al., 2011; Wang F. Z. et al., 2022).

Capsaicinoids accumulation is determined by genotype or cultivar, node position, fruiting and maturity stages, and environmental growth conditions such as light, temperature, water, and mineral nutrition (Naves et al., 2019; Uarrota et al., 2021). The degree of pungency is variable between different *Capsicum* cultivars. The 12 different varieties of *Capsicum* cultivars belonging to three species (*Capsicum annuum*, *Capsicum chinense*, and *Capsicum frutescens*) showed considerable variation in capsaicinoids content (Giuffrida et al., 2013). The biosynthesis of capsaicinoids is related to the convergence of the phenylpropanoid and branched-chain fatty acid pathways, which involve three nitrogenous molecules: phenylalanine, valine, and leucine (Bennett and Kirby, 1968; Leete and Louden, 1968). Currently, the raw materials for industrial production of capsaicinoids are mainly derived from the fruit, which is high cost and low yield (Zhu et al., 2019). It is necessary to improve capsaicinoids yield. Many studies have been reported that the capsaicinoids content in *Capsicum* fruits has been associated positively with available N (Medina-Lara et al., 2008; Aldana-Iuit et al., 2015; Zhang et al., 2020). Some reports have shown that microorganisms can promote plant growth and the accumulation of metabolites (Lv et al., 2024). The inoculation of pepper fruit with *Streptomyces pactum* Act12 has been demonstrated to increase the content of vitamins, phenolic acids, alkaloids, and flavonoids (Zhao et al., 2023). To date, there have been no reports of the use of microorganisms to increase capsaicinoids content in pepper.

The rhizosphere, the soil adjacent to roots, provides a niche for the interaction between plant roots and microorganisms (Singh et al., 2004; Philippot et al., 2013). The rhizosphere microbiomes provide a number of beneficial functions for plant host, including growth promotion, nutrient uptake, stress tolerance and resistance to pathogens. So, it is also referred to as the plant’s second genome, and is crucial for plant health (Berendsen et al., 2012). Plant secondary metabolites play an important role in the interaction between plants and microorganisms, which can protect plants against microbial pathogens (Cipollini, 2000). Recent studies have shown that plant secondary metabolites affect microbiome composition and function. Prominent among those metabolites are the glucosinolates (Siebers et al., 2018), flavonoids (Kudjordjie et al., 2021), coumarins (Stringlis et al., 2018; Voges et al., 2019), benzoxazinoids (Hu et al., 2018; Cotton et al., 2019; Kudjordjie et al., 2019), and triterpenes (Huang et al., 2019). Benzoxazinoids are indole-derived compounds exuded by the roots (de Bruijn et al., 2018). Three reports reveal that maize mutants unable to synthesize BXs showed significant alterations in composition of both bacterial and fungal communities (Hu et al., 2018; Cotton et al., 2019; Kudjordjie et al., 2019). The phenolic compounds coumarins are relatively abundant in the rhizosphere, and have function in iron acquisition. Two recent studies have shown that coumarins also play a key role in shaping the root microbiome composition, as shown by analyses of microbiota assemblies of coumarin-deficient *Arabidopsis* mutants (Stringlis et al., 2018; Voges et al., 2019). In terms of the triterpenes thalianin and arabidin, these two metabolites contribute substantially to the assembly of *Arabidopsis*-specific root microbiota (Huang et al., 2019). It is worth noting that many metabolites shown to affect plant microbiome also trigger changes in the composition of gut microbiome. These include the glucosinolates (Kellingray et al., 2017), terpenoids (Chen et al., 2021), flavonoids (Baky et al., 2022), and phenolic compounds (Domínguez-Avila et al., 2021).

Capsaicinoids is a kind of secondary metabolite from pepper, and many evidences suggest that capsaicinoids can influence the composition, abundance, and function of the gut microbiota (Baboota et al., 2014; Wang et al., 2020). Moreover, capsaicinoids concentration influence microbial communities and kimchi metabolites during kimchi fermentation (Park et al., 2019). Yet, whether capsaicinoids affect plant microbiome has not yet been addressed. In addition, the effects of rhizosphere microbiota on root metabolite composition and exudation have also been reported. Korenblum et al. (2020) showed that different microbial communities induce systemic changes in metabolite exudation of tomato root. For instance, bacteria affiliated with the genus *Bacillus* induced synthesis of acylsugars secondary metabolites. However, the relationship between rhizosphere microorganisms and the level of capsaicinoids is unknown. It has been reported that plant genotype and soil type have a significant impact on their rhizosphere microbiomes (Berg and Smalla, 2009; Lundberg et al., 2013; Yeoh et al., 2017; Veach et al., 2021). Soil available nutrient concentrations dominate the assembly of rhizosphere bacterial community (Ren et al., 2020). Furthermore, differences between plant genotypes influence the composition and function of the rhizosphere microbiomes in many plants, such as rice (Hardoim et al., 2013), corn (Peiffer et al., 2013), wheat (Meyer et al., 2010), potato (Weinert et al., 2011), and soybean (Zhong et al., 2019). However, it is largely lacking how pepper varieties affect the structure and functions in rhizosphere bacterial and fungal communities.

Here, we collected rhizosphere soil from five pepper varieties with different pungency, to analyze the rhizosphere fungal and bacterial communities by 16S rRNA gene and ITS amplicon sequencing technologies. The objective of this study was to investigate the effect of pepper varieties and its capsaicinoids content on the rhizosphere microbial community. We demonstrated that pepper variety and its capsaicinoids content significantly influenced the structure and composition of rhizosphere communities under field conditions, which may affect the biosynthesis of capsaicinoids. This will help us understand the influence of the pepper genotype and trait on the interaction between microorganisms and plants.

## Materials and methods

### Plant materials and field trials

Seeds of five pepper varieties, XY6, XY21, XY39, XY40, and XY42, were obtained from Hunan Xiangyan Seed Industry Co., Ltd. All of them belong to *Capsicum annuum*, except XY40, which is a hybrid line of *Capsicum annuum* and *Capsicum chinense*. The surface-sterilized seeds were sown in nutrient substrate. At eight leaf stage, the seedlings were transplanted to the experimental field. Field trials were performed from April to August in 2021 at the Gaoqiao experimental field of Hunan Xiangyan Seed Industry CO., LTD. Samples of pepper rhizosphere soil and pepper fruit were collected at the mature green stage. Four biological duplicates were obtained for each pepper variety, either soil or fruit samples. Soil samples were collected using the root shaking method (Inceoǧlu et al., 2010). Each soil sample was divided into two parts, one part of was stored at 4°C for physicochemical properties, the other part was stored at −80°C for sequencing analysis. The placenta tissues were isolated from fruits for capsaicinoids content determination (Tanaka et al., 2017).

### Determination of capsaicinoids contents

The dried placental tissues were powdered and 0.1 g were treated with 20 mL acetonitrile using an ultrasonic machine at the temperature of 65°C for 20 min. After low-speed centrifugation, the extract solution was filtered through 0.2 μm filters (Millipore, USA). The analysis of capsaicinoids were performed by high-performance liquid chromatography (HPLC, LC-20AT, SHIMADZU, Japan). The capsaicin (C) and dihydrocapsaicin (DHC) standards or extracted samples were injected into a Shim-pack GIST C18 column (dimension 250 × 4.6 mm, particle size 5 μm). An isocratic mixture of water: acetonitrile was used as the mobile phases. The injection volume, flow rate, run time, and temperature were 10 μL, 1.0 mL/min, 30 min, and 30°C, respectively. The C and DHC were detected with the UV detector at 222 nm. The capsaicinoids content (CAPs) was calculated as (C + DHC)/0.91 (Bennett and Kirby, 1968).

### Soil physicochemical analysis

Soil pH was measured in water with a soil/water ratio of 1:2.5 (w/v) by a FE20 pH meter (Mettler-Toledo International Inc., China) (Liu et al., 2020). Soil available nitrogen (AN) was determined using the alkaline hydrolysis diffusion method (Hu et al., 2008). Soil available phosphorus (AP) and available potassium (AK) were tested according to methods described by Fan et al. (2019). Soil organic matter (OM) was determined using the K_2_CrO_7_-H_2_SO_4_ method (Bremner and Jenkinson, 1960).

### 16S rDNA and ITS sequencing

Total DNA from rhizosphere soil samples was extracted using the E.Z.N.A.^®^ Soil DNA Kit (Omega, Inc., USA) according to the manufacturer’s instructions. DNA concentration and purity were determined with NanoDrop 2000 spectrophotometer (Thermo Fisher Scientific, CA, USA). The primer pairs 341F/805R (5′-CCTACGGGNGGCWGCAG-3′/5′-GACTACHVGGGTATCTAATCC-3′) and ITS1FI2/ITS2 (5′-GTGARTCATCGAATCTTTG-3′/5′-TCCTCCGCTTATTGATATGC-3′) were used to amplify the bacterial 16S V3-V4 region and fungal ITS2 region, respectively. The PCR products were confirmed with 2% agarose gel electrophoresis and purified by AMPure XT beads (Beckman Coulter Genomics, Danvers, MA, USA) and quantified by Qubit (Invitrogen, USA). Sequencing was performed at S LC-Bio Technologies (Hangzhou, China) Co., Ltd., using an Illumina NovaSeq platform.

### Bioinformatic analysis

Paired-end reads was assigned to samples based on their unique barcode and truncated by cutting off the barcode and primer sequence. Paired-end reads of 16S rRNA gene and ITS gene were merged using FLASH version 1.2.8 (Magoč and Salzberg, 2011) and PEAR version 0.9.6 (Zhang et al., 2014), respectively. Quality filtering on the raw reads were performed under specific filtering conditions to obtain the high-quality clean tags according to the FQTRIM version 0.94 (Pertea, 2018). Chimeric sequences were filtered using VSEARCH software version 2.3.4 (Rognes et al., 2016). After dereplication using DADA2 (Callahan et al., 2016), we obtained feature table and feature sequence. Taxonomic assignment of 16S rRNA gene and ITS gene was performed using QIIME2 plugin feature-classifier against the SILVA v132 database (Quast et al., 2013) and UNITE 8.2 database (Nilsson et al., 2019), respectively. The relative abundance (X bacteria or fungi count/total count) is used in bacteria or fungi taxonomy. Alpha diversity and Beta diversity were analyzed by QIIME2 process (Bolyen et al., 2019), and pictures were drawn by R package (v3.5.2) (Dixon, 2003). Alpha diversity included Chao1 and Shannon indices. Beta diversity was calculated using unweighted UniFrac distance (Lozupone et al., 2007) and visualized through a principal coordinate analysis (PCoA) (Ramette, 2007). Analysis of similarities (ANOSIM) was carried out using R (version: 3.4.3) and the Vegan package in R (version: 2.3.0). The R value and *p* value of the ANOSIM were calculated using a permutation test with 999 permutations. Linear discriminant analysis effect size (LEfSe) analysis was performed using the OmicStudio tools^1^ (Kruskal–Wallis test *p* < 0.05, and LDA score > 3.5) (Segata et al., 2011). Spearman’s rank correlation coefficients analysis was also performed using the OmicStudio tools (|*r*| > 0.5 and *p* < 0.05).^2^

### Statistical analysis

Means and standard deviation values were calculated using SPSS software version 19.0. Tukey’s multiple comparison testing was used to evaluate the significant differences among varieties. Figures were constructed in GraphPad Prism version 8.0 (GraphPad Software Inc., San Diego, CA, USA).

## Results

### Capsaicinoids content of different pepper varieties

**Figure 1:**
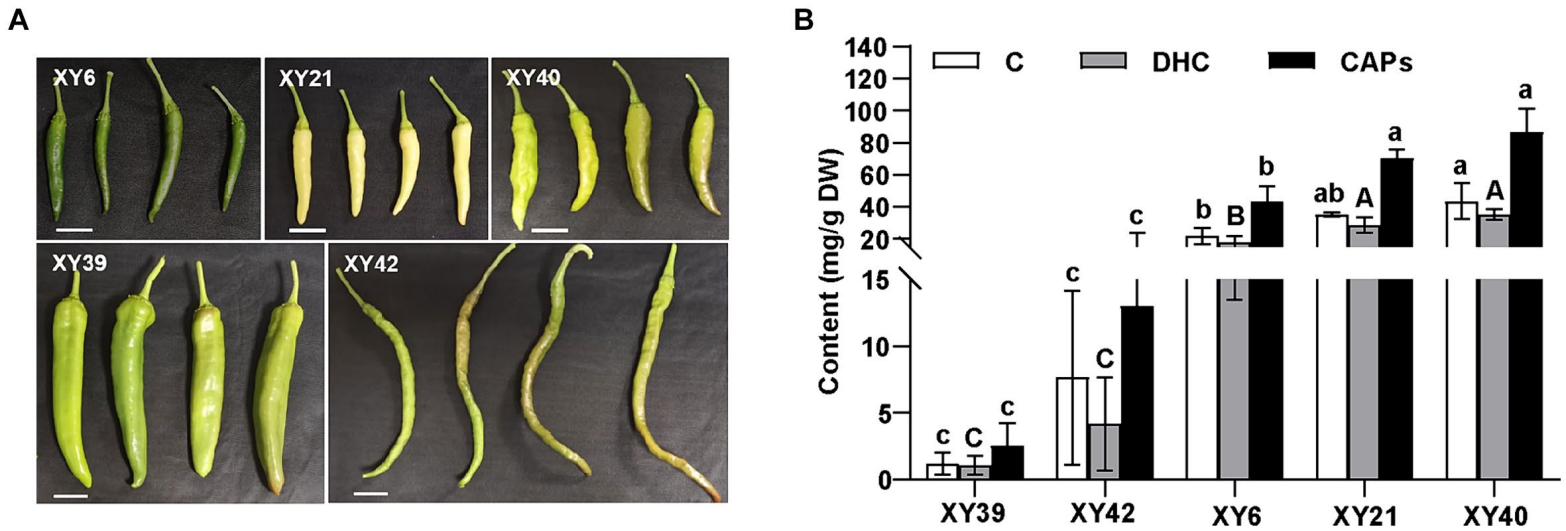
The capsaicinoids accumulation of pepper at the mature green stage. **(A)** The phenotypes of fruits of five pepper varieties. **(B)** The capsaicin (C), dihydrocapsaicin (DHC) and capsaicinoids (CAPs) contents in pepper placenta. Tukey’s multiple comparisons tests were performed to identify the significant differences; the same letter indicates a nonsignificant difference between the means (*p* > 0.05).

Five varieties of pepper, XY6, XY21, XY39, XY40 and XY42, were used in this study (Figure 1A). The capsaicinoids contents (CAPs) were detected in the placenta of the five varieties (Figure 1B). XY40 and XY21 contained the highest capsaicinoids content (86.54 mg/g and 70.2 mg/g) in the placenta, and showed significantly higher capsaicinoids than XY6 (43.41 mg/g). Meanwhile, XY40, XY21 and XY6 showed significantly higher capsaicinoids than XY42 (12.99 mg/g) and XY39 (2.53 mg/g). The contents of capsaicin (C) and dihydrocapsaicin (DHC) in the placenta were in line with that of capsaicinoids.

### Soil physicochemical properties

The physicochemical characteristics of the soil from different pepper varieties were summarized in Table 1. The pH of the five different soils ranged from 4.9 to 5.3, showing no significant differences. The AK and AP contents of soils from XY6 and XY21 markedly increased compared to that of XY39 and XY42. The soil from XY6 exhibited significantly OM and AN contents compared to soils from XY21, XY40, XY39 and XY42.

**Table 1:** Basic physical–chemical properties of rhizosphere soil.

Group	pH	Organic matter g·kg^−1^	Available N mg·kg^−1^	Available P mg·kg^−1^	Available K mg·kg^−1^
XY39	5.3 ± 0.3a	30.3 ± 1.0b	147.9 ± 3.1b	111.1 ± 10.7c	120.6 ± 9.6c
XY42	5.3 ± 0.3a	28.4 ± 0.6b	136.9 ± 5.3b	132.1 ± 30.8bc	126.6 ± 25.5c
XY6	4.9 ± 0.4a	34.4 ± 1.4a	198.5 ± 20.9a	227.3 ± 30.7a	304.6 ± 71.6a
XY21	5.1 ± 0.4a	30.8 ± 0.7b	156.9 ± 7.5b	175.2 ± 31.7ab	213.8 ± 42.0b
XY40	5.3 ± 0.1a	30.7 ± 2.8b	152.4 ± 13.2b	112.0 ± 20.8c	137.4 ± 9.0bc

Data represent means ± SD. Different letters indicate significant differences among the five varieties in Tukey’s multiple comparisons test.

### Diversity of microbial community in rhizosphere soil of different pepper varieties

A total of 1,474,543 raw bacterial sequences and 1,689,023 fungal sequences were identified using Illumina MiSeq analysis. After a series of preprocessing steps, 1,158,561 bacterial and 1,633,413 fungal qualified reads were classified into 19,459 bacterial and 2,799 fungal operational taxonomic units (OTUs), respectively. Among them, 6,715 (34.51%) bacterial OTUs and 1,712 (61.16%) fungal OTUs failed to be identified to any known phylum based on the UNITE database. The amplicon sequence data for the 16S and ITS in the paper have been uploaded to the NCBI Sequence Read Archive (SRA) database with accession number PRJNA1145089.

**Figure 2:**
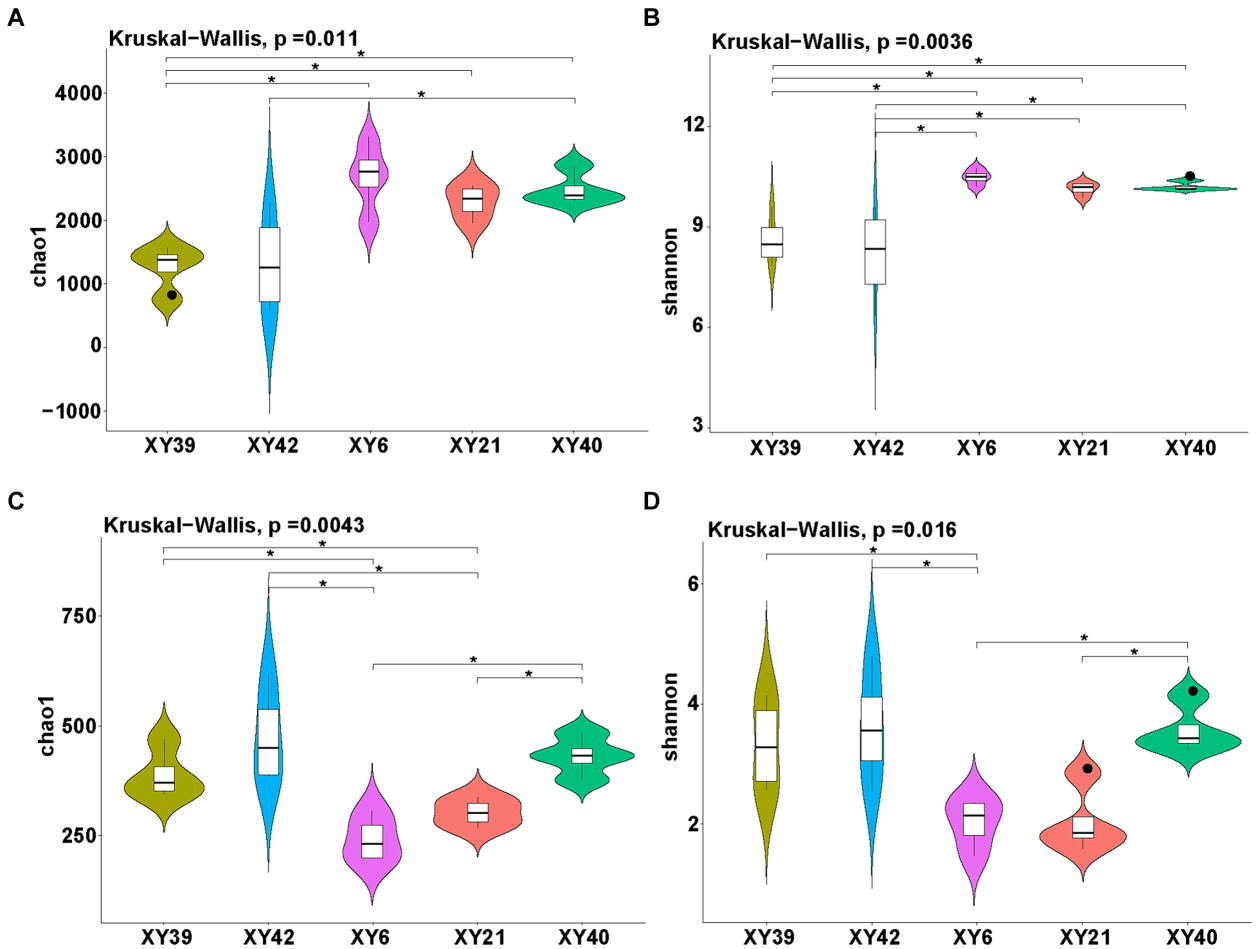
The diversity evaluation of rhizosphere microbial communities of five pepper varieties. **(A,B)** Comparison of bacterial communities among five varieties using the Chao1 index **(A)** and Shannon index **(B)**. **(C,D)** Comparison of fungal communities among five varieties using the Chao1 index **(C)** and Shannon index **(D)**. Asterisk indicates significant differences (*p* < 0.05).

Alpha diversity of rhizosphere microbial community of pepper was estimated by both the Chao1 index (richness) and Shannon index (diversity). The results showed that the richness and diversity of bacteria and fungi in rhizosphere soil exhibited significantly differences among different pepper varieties (Figure 2). Specifically, for bacteria, the Shannon index of XY6, XY21 and XY40 was significantly higher than that of XY39 and XY42, whereas the Chao1 index in XY6, XY21 and XY40 was significantly higher than in XY39 alone. Moreover, the Chao1 index of XY40 was significantly higher than that of XY42 (Figures 2A,B). In terms of the fungal community, the Chao1 index of XY6 and XY21 was significantly lower than that of XY39 and XY42, whereas the Shannon index in XY6 alone was significantly lower than in XY39 and XY42 (Figures 2C,D), which suggested that fungal richness and diversity were contrary to bacterial richness and diversity among different pepper varieties.

**Figure 3:**
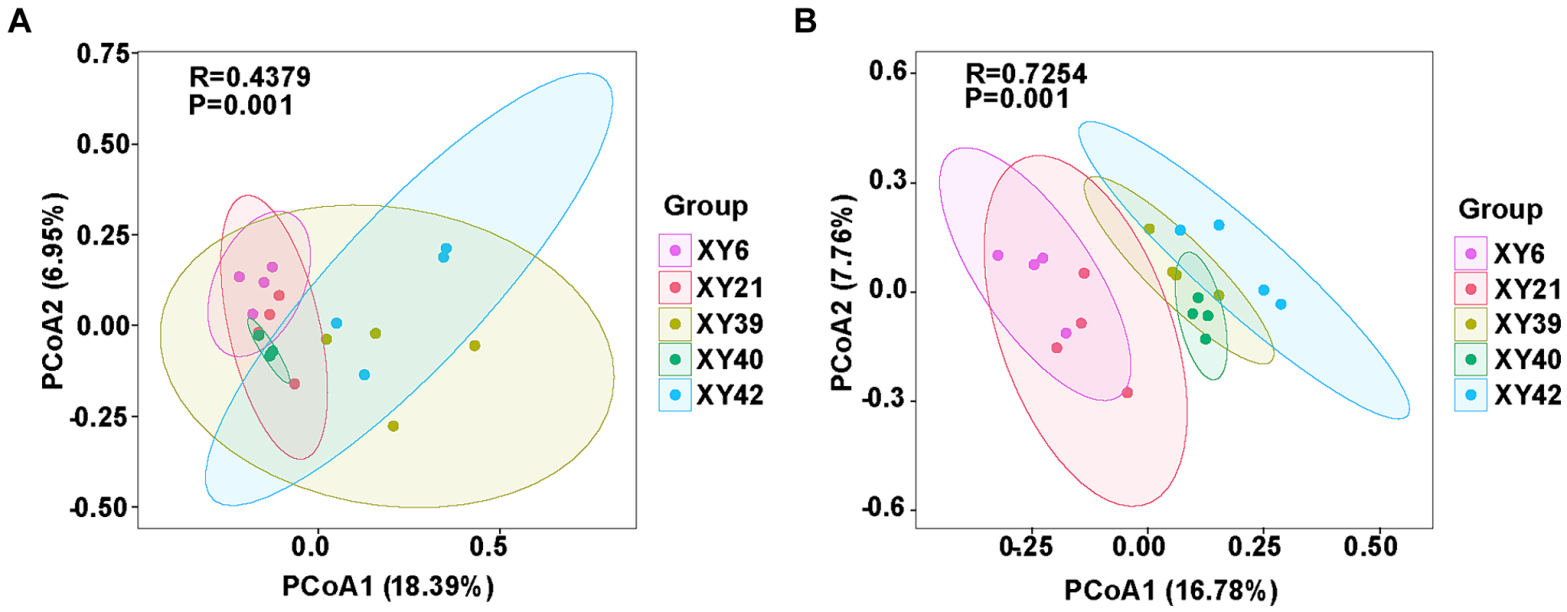
The comparison of rhizosphere microbial communities [**(A)** bacteria; **(B)** fungi] among five pepper varieties using PCoA and ANOSIM. PCoA, principal coordinate analysis; ANOSIM, analysis of similarities.

Beta diversity of rhizosphere microbial community of pepper was also calculated by unweighted UniFrac distance. PCoA revealed significant variations in the bacterial and fungal communities among the different varieties. PCoA1 and PCoA2 of the bacterial community explained 18.39 and 6.95% of the variations, respectively (Figure 3A). Meanwhile, PCoA1 and PCoA2 of the fungal community explained 16.78 and 7.76% of the variations, respectively (Figure 3B). Furthermore, results from ANOSIM comparisons demonstrated that varieties had significant effects on the bacterial community (*R* = 0.4379; *p* = 0.001) and fungal community (*R* = 0.7254; *p* = 0.001) (Figure 3).

### Microbial community composition in rhizosphere soil of different pepper varieties

**Figure 4:**
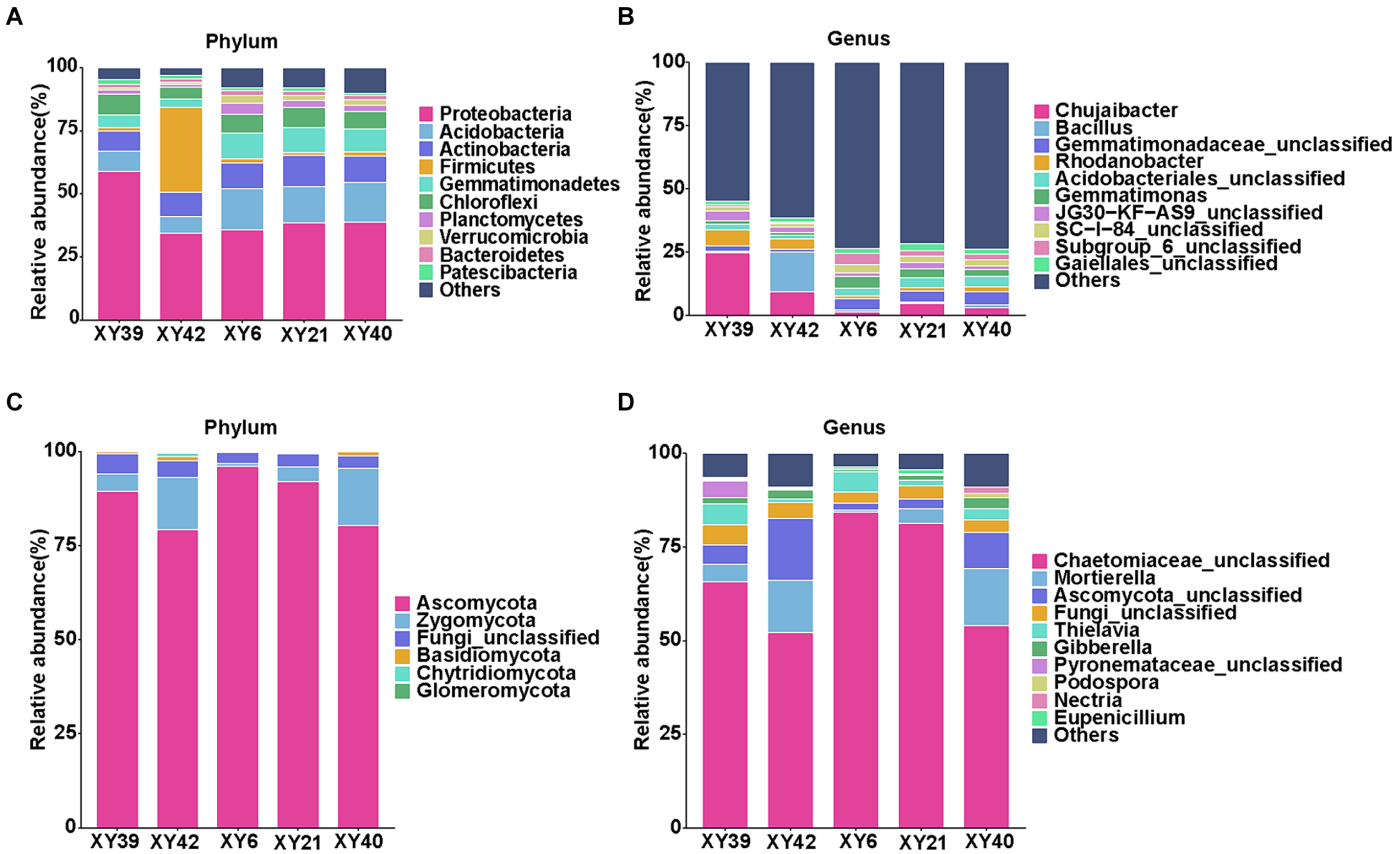
Community composition in the rhizosphere soil of five varieties. **(A,B)** Relative abundances of bacterial phylum **(A)** and genus **(B)**. **(C,D)** Relative abundances of fungal phylum **(C)** and genus **(D)**.

At the phylum level, the dominant bacterial phyla of rhizosphere soils across all varieties were *Proteobacteria*, *Acidobacteria*, *Actinobacteria*, *Firmicutes*, and *Gemmatimonadetes* (Figure 4A). *Proteobacteria* accounted for the most significant proportion reaching to 32.93–55.46% of all OTUs. The dominant fungal phyla of rhizosphere soils across all varieties were *Ascomycota* and *Zygomycota* (Figure 4C). In term of the genus level, the most abundant bacterial genera were *Chujaibacter*, *Bacillus*, *Gemmatimonadaceae*_*unclassified, Rhodanobacter*, *Acidobacteriales_unclassified*, and *Gemmatimonas* (Figure 4B), and the predominant fungal genera were *Chaetomiaceae_unclassified*, *Mortierella*, *Ascomycota_unclassified*, and *Thielavia* in all varieties (Figure 4D).

Then we compared the differences in bacterial phyla and genera between rhizosphere soils of five varieties. At the phylum level, *Acidobacteria*, *Gemmatimonadetes*, and *Verrucomicrobia* were significantly more abundant in XY6, XY21 and XY40 than XY39 and XY42 (*p* < 0.05, Figure 4A). The relative abundance of *Firmicutes* in XY42 was significantly higher than that of the other varieties (*p* < 0.01, Figure 4A). At the genus level, the relative abundance of *Chujaibacter* and *Rhodanobacter* was significantly decreased in XY6, XY21 and XY40 compared to XY39 and XY42. The relative abundance of *Gemmatimonas* in XY6 and XY21 was significantly higher than that in XY39 and XY42 (*p* < 0.05, Figure 4B). The relative abundance of *Bacillus* in XY42 was significantly higher than that in other varieties (*p* < 0.01, Figure 4B). Next, we compared the differences in fungal phyla and genera between rhizosphere soils of five varieties. It was found that *Zygomycota* was the phylum with higher relative abundance in XY42 than that in XY6 and XY21, and *Glomeromycota* is unique to XY42 (*p* < 0.01, Figure 4C). *Mortierella* and *Gibberella* were the genera with lower relative abundance in XY6 than that in other varieties (*p* < 0.05, Figure 4D). Compared with bacteria, the fungal differences were not particularly significant.

**Figure 5:**
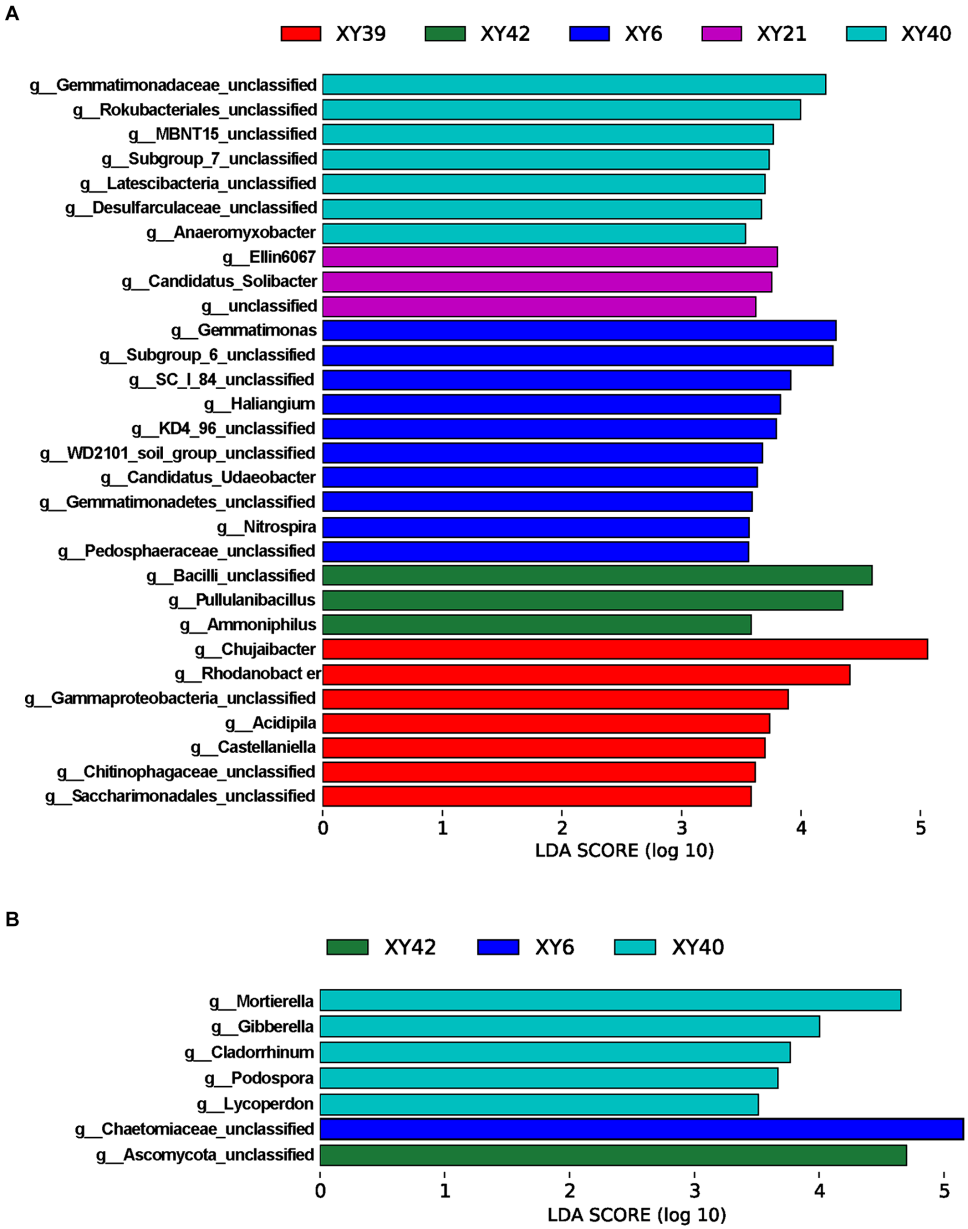
LDA scores of the genera biomarkers in the rhizosphere soil of five pepper varieties. **(A)** Bacteria. **(B)** Fungi. LDA scores are shown as horizontal bars for the genus biomarkers with an LDA score > 3.5 as listed, Kruskal–Wallis rank sum test, *p* < 0.05. LDA, linear discriminant analysis.

To further screen the rhizosphere bacteria and fungi affected by variety, the LEfSe was employed to identify biomarkers among varieties at the genus level. The results showed that there were 30 bacterial biomarkers and 7 fungal biomarkers in the rhizosphere soil of the five varieties (Figure 5). In XY39, the main bacterial biomarkers were *Chujaibacter* and *Rhodanobacter*. In XY42, *Pullulanibacillus* and *Ammoniphilus* were the main bacterial biomarkers. In XY6, *Gemmatimonas*, *Haliangium* and *Nitrospira* were the prominent bacterial biomarkers. In XY21, the genera that differed significantly from other varieties were *Ellin6067* and *Candidatus_Solibacter* (Figure 5A). For XY40, the main bacterial biomarker was *Anaeromyxobacter*, and fungal biomarkers were *Mortierella*, *Gibberella*, and *Podospora* (Figures 5A,B). These results suggested that varieties markedly impacted bacterial and fungal communities in pepper.

### Relationships between soil factors, capsaicinoids content and microbial communities

**Figure 6:**
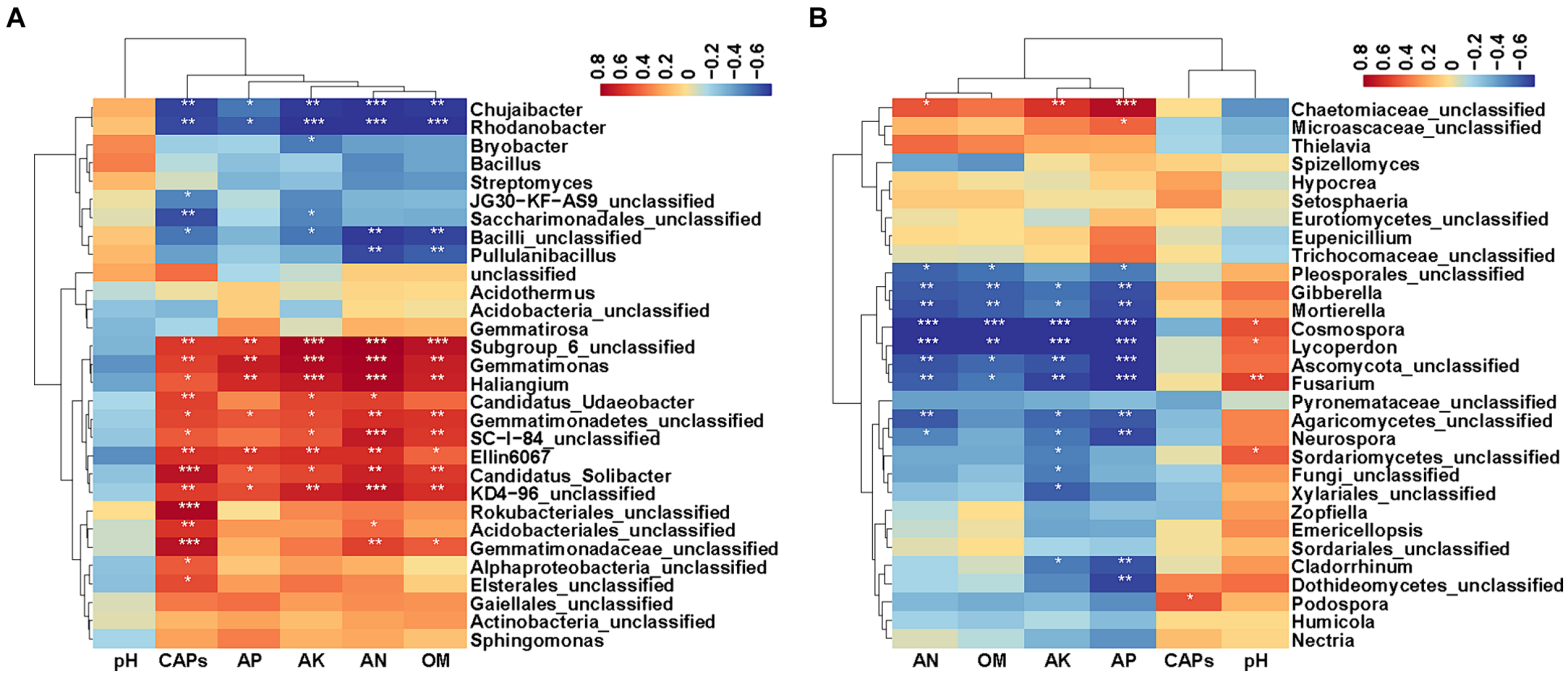
Heatmaps of the Spearman correlation coefficient. **(A)** Heatmap of the correlation between soil factors, capsaicinoids content and the top 30 abundant bacterial genera. **(B)** Heatmap of the correlation between soil factors, capsaicinoids content and the top 30 abundant fungal genera. Asterisks indicate significant differences, *p < 0.05; **p < 0.01; ***p < 0.001.

To explore relationships between soil factors (shown in Table 1), capsaicinoids content (CAPs) and bacterial and fungal composition at the genus level (top 30 microbial genera), Spearman correlation analysis was carried out (Figure 6). The results showed that CAPs, AP, AK, AN and OM had significant effects on bacteria and fungi. The top 30 bacterial genus clusters could be divided into five subclusters. Subcluster 1 and subcluster 2 were negatively correlated with CAPs, AP, AK, AN and OM. Subcluster 3 and subcluster 4 were positively correlated with CAPs, AP, AK, AN and OM. Specially, *Ellin6067*, *Gemmatimonas*, *Haliangium*, and *Candidatus_Solibacter* were significantly positively correlated with CAPs, AP, AK, AN and OM, whereas *Chujaibacter* and *Rhodanobacter* were significantly negatively correlated with CAPs, AP, AK, AN and OM. *Candidatus_Udaeobacter* was significantly positively correlated with CAPs, AK and AN. *Pullulanibacillus* was significantly negatively correlated with AN and OM, and *Bryobacter* was significantly negatively correlated with AK (Figure 6A, *p* < 0.05).

The top 30 fungal genus clusters could also be divided into five subclusters (Figure 6B). Subcluster 1 was positively correlated with AP, AK, AN and OM, but it was negatively correlated with pH. Subcluster 3 and subcluster 4 were negatively correlated with AP, AK, AN and OM, and positively correlated with pH. Specially, *Gibberella*, *Mortierella*, *Cosmospora*, *Lycoperdon*, and *Fusarium* were significantly negatively correlated with AP, AK, AN and OM. *Cosmospora*, *Lycoperdon*, and *Fusarium* were significantly positively correlated with pH. *Neurospora* was significantly negatively correlated with AP, AK, AN and *Cladorrhinum* was significantly negatively correlated with AP and AK. In addition, *Podospora* showed a significant positive correlation with CAPs (Figure 6B, *p* < 0.05).

## Discussion

The interactions between roots and their rhizosphere microbiota are critical to plant growth and performance (Lu et al., 2018; Kwak et al., 2018). Root microbiota drive plant growth (Qu et al., 2020) and play a significant role in nutrient acquisition (Chibucos and Tyler, 2009) and soil nutrient dynamics (Hou et al., 2018). Currently, multiple reports demonstrated that the rhizosphere microbial communities are genetically regulated by host genotype and related traits (Bulgarelli et al., 2012; Bouffaud et al., 2014; Lebeis et al., 2015). At present, studies of the effect of capsaicin on microbial communities have particular focused on gut microbiota (Baboota et al., 2014; Wang et al., 2020) and pepper fermentation (Shi et al., 2023). There is currently very little evidence of the effect of capsaicin on plant inter-root microbial communities. In this study, five pepper varieties with different pungency were investigated for influences of genotypes on rhizosphere microbial communities. The Chao1 and Shannon indices revealed that there were differences in the richness and diversity of rhizosphere microorganisms among different pepper varieties (Figure 2). Furthermore, both PCoA and ANOSIM results showed that pepper varieties had significant effects on the structure of rhizosphere microorganisms (Figure 3). Those findings were in agreement with previous studies, which revealed that there were significant differences in rhizosphere microbial communities among different potato, tea and alfalfa varieties (Marques et al., 2014; Zhang et al., 2022; Qi et al., 2023). Moreover, the richness and diversity of the bacteria in XY40, XY21 and XY6 were significantly higher than that in XY39 and XY42 (Figure 2A), and XY40, XY21 and XY6 also exhibited significantly higher CAPs than XY42 and XY39 (Figure 1B), suggesting that CAPs may increase bacterial richness and diversity. However, similar results were not obtained for fungal. Only for XY6, the richness and diversity of fungal were different and were significantly lower compared to XY42 and XY39 (Figure 2B), indicating that CAPs may decrease fungal richness and diversity. These results demonstrated that bacteria and fungi showed different response to CAPs. Capsaicin has been reported to have antifungal activity against pathogens (Vuerich et al., 2023). We hypothesize that this is related to the fact that capsaicin has an antifungal effect, and that the higher the capsaicin content, the lower the abundance of the fungus, leading to this result. Side by side, this illustrates the potential of capsaicin as a bio-antifungal agent.

*Proteobacteria*, *Acidobacteria*, and *Actinobacteria* were the dominant bacterial phyla in rhizosphere of pepper (Figure 4A), which was consistent with previous studies on maize (Kong et al., 2019; Li et al., 2022) and *Vaccinium* cultivated soils (Yurgel et al., 2017; Li et al., 2020; Ramirez-Villacis et al., 2023). This result was expected because *Proteobacteria* members are well adapted to plant rhizosphere across diverse plant species (Philippot et al., 2013; Yang et al., 2023), and *Acidobacteria* is one of the most abundant bacterial phyla in the rhizosphere soil (Lee et al., 2008; Cheng et al., 2020). For fungal community, the detected fungal phyla were dominantly composed of *Ascomycota* in rhizosphere (Figure 4C), as reported in previous studies (Swift et al., 2021; Zahid et al., 2021; Darriaut et al., 2022; Marasco et al., 2022; Qi et al., 2023). The composition of rhizosphere community was also different among different pepper varieties (Figure 4). Our study found that *Acidobacteria*, *Gemmatimonadetes* and *Verrucomicrobia* were significantly higher in the rhizosphere soil from XY40, XY21 and XY6 than XY39 and XY42 (Figure 4A). Interestingly, XY40, XY21 and XY6 also exhibited significantly higher CAPs than XY42 and XY39 (Figure 1B). Therefore, we hypothesized that the difference in CAPs content may be the reason for the differences in rhizosphere bacterial community composition among different varieties. *Acidobacteria*, *Gemmatimonadetes* and *Verrucomicrobia* belong to oligotrophic bacteria. According to oligotrophic-copiotrophic theory, oligotrophic bacteria can maintain growth and be prevalent under nutrient-poor conditions. In contrast, copiotrophic bacteria (e.g., *Proteobacteria* and *Firmicutes*) usually are more prevalent in nutrient-rich conditions (Fierer et al., 2007). In our results, the nutrient contents (AK and AP) of soils from XY6 and XY21 were higher compared to soils from XY39 and XY42 (Table 1), which was contrary to the oligotrophic-copiotrophic theory. Additionally, the copiotrophic phylum *Firmicutes* also did not follow the predicted patterns (Figure 4A), suggesting that the differences in bacterial community composition of pepper were not due to soil nutrient contents. These conclusions validated our hypothesis that the changes in rhizosphere bacterial community composition of different pepper varieties were at least partially due to CAPs content. For fungal community, only phylum *Zygomycota* in XY42 with lower CAPs content was higher than in XY21 and XY6 with higher CAPs content (Figure 4C). The changes were not significant between different varieties, which suggested that CAPs had a greater effect on the bacterial community than fungal community.

Many bacterial taxa responded to CAPs, whereas less fungal taxa responded to CAPs. For example, seven bacterial genera, including three predominant bacterial genera (*Gemmatimonas*, *Chujaibacter* and *Rhodanobacter*), were significantly correlated with CAPs (Figure 6A). For the fungi, only *Podospora* showed a significant correlation with CAPs (Figure 6B). Moreover, the relative abundance of the three predominant bacterial genera were significantly different between high CAPs varieties (XY21 and XY6) and low CAPs varieties (XY39 and XY42) (Figure 4B). However, the relative abundance of *Podospora* was only significantly higher in XY40 (high CAPs) than in XY42 (low CAPs) (Figure 4D). These results further supported our hypothesis that CAPs affected microbial communities among different pepper varieties, and demonstrated the relative differences in response to CAPs of bacteria versus fungi. In addition, the seven bacterial genera were also significantly correlated with soil factors such as AP, AK, AN and OM (Figure 6A), demonstrating both CAPs and soil factors might be the reasons for the change of bacterial community, however, which factor has a greater effect on bacterial community needs to be further verified. In terms of fungal genera, *Mortierella* and *Gibberella* were significantly negatively correlated with AP, AK, AN and OM but not CAPs (Figure 6B). Furthermore, compared with other varieties, the relative abundance of *Mortierella* and *Gibberella* were lower in XY6 with the highest soil nutrient contents (AP, AK, AN and OM) (Figure 4D). However, similar results were not obtained in bacteria. These results indicated that fungal communities were more susceptible to soil environment compared to bacterial communities in the rhizosphere of pepper, which was consistent with the results in young grapevines (Darriaut et al., 2022), but contrary to the results of the study on blueberries was (Ramirez-Villacis et al., 2023).

A large number of studies have found that differences in the rhizosphere microbiome led to differences in host performance (Badri et al., 2013; Wagner et al., 2014; Fitzpatrick et al., 2018). So, we speculated that rhizosphere microbial communities can influence the biosynthesis of CAPs. Our study found that *Ellin6067*, *Gemmatimonas*, *Candidatus_Udaeobacter*, and *Candidatus_Solibacter* were significantly positively correlated with both AN and CAPs (Figure 6A). *Ellin6067 and Candidatus_Solibacter* belong to the phylum *Acidobacteria*, *Gemmatimonas* belongs to the phylum *Gemmatimonadetes*, and *Candidatus_Udaeobacter* belongs to the phylum *Verrucomicrobia* (Schoch et al., 2020). At phylum level, *Acidobacteria*, *Gemmatimonadetes* and *Verrucomicrobia* were significantly more abundant in high CAPs varieties (XY40, XY21 and XY6) than low CAPs varieties (XY39 and XY42) (Figure 4A). *Acidobacteria* and *Gemmatimonadetes* had the function of nitrogen fixation (Li et al., 2023). *Candidatus_Udaeobacter* was considered to be the potential bacteria taking part in nitrogen transformation (Gao et al., 2022). On the contrary, *Chujaibacter* and *Rhodanobacter* were significantly negatively correlated with both AN and CAPs (Figure 6A). It was reported that *Rhodanobacter* had the ability of denitrification (Prakash et al., 2012). *Chujaibacter* was also considered to be the predominant population involved in nitrogen removal (Xin et al., 2021). N availability in soils directly affected CAPs production (Medina-Lara et al., 2008; Aldana-Iuit et al., 2015; Zhang et al., 2020). These conclusions supported our hypothesis that these bacteria might regulate biosynthesis of CAPs by increasing the N availability of soils. In addition, almost all bacterial genera that significantly correlated with CAPs content could be found in the biomarkers list analyzed by LEfSe (Figure 5A). *Chujaibacter* and *Rhodanobacter*, with a negative correlation with CAPs, corresponded to the biomarkers of XY39 with a lower CAPs content, while *Gemmatimonas*, *Haliangium, Ellin6067* and *Candidatus_Solibacter*, with a positive correlation with CAPs, corresponded to the biomarkers of XY6 and XY21 with a higher CAPs content (Figures 1B, 5A, 6A). Previous studies have found that there were differences in the rhizosphere bacterial community structure between local varieties and artificially selected strains of pepper (Wang C. et al., 2022; Wang et al., 2023), which was consistent with our findings, but more importantly, this study is the first to demonstrate that CAPs are also the main factor affecting the rhizosphere microbial community among different pepper varieties.

In summary, we analyzed the effects of different pepper varieties on rhizosphere microbial community, and explored the role of rhizosphere microorganisms in improving CAPs content of pepper. The results reported here indicate that pepper variety significantly impacts the structure and composition of pepper rhizosphere microbial communities. Changes in microbial communities were significantly associated with CAPs among different pepper varieties, and the bacterial communities were more impacted by the CAPs than the fungi. These findings help to better understand the complex interactions between host plants and microbiome. It also offers the possibility of manipulating the plant rhizosphere microbiome as an effective strategy to improve CAPs content of pepper.

## Data Availability

The original contributions presented in the study are included in the article/supplementary material, further inquiries can be directed to the corresponding authors.

## Footnotes

^1^ https://www.omicstudio.cn/tool/

^2^ https://www.omicstudio.cn
